# Detecting and staging podoconiosis cases in North West Cameroon: positive predictive value of clinical screening of patients by community health workers and researchers

**DOI:** 10.1186/s12889-016-3669-6

**Published:** 2016-09-20

**Authors:** Samuel Wanji, Jonas A. Kengne-Ouafo, Fabrice R. Datchoua-Poutcheu, Abdel Jelil Njouendou, Dizzel Bita Tayong, David D. Sofeu-Feugaing, Nathalie Amvongo-Adjia, Bridget A. Fovennso, Yolande F. Longang-Tchounkeu, Fasil Tekola-Ayele, Peter A. Enyong, Melanie J. Newport, Gail Davey

**Affiliations:** 1 Parasites and Vector Biology research unit (PAVBRU), Department of Microbiology and Parasitology, University of Buea, Buea, Cameroon; 2 Research Foundation for Tropical Diseases and the Environment (REFOTDE), Buea, Cameroon; 3 Department of Biochemistry and Molecular Biology, University of Buea, Buea, Cameroon; 4 Laboratory of Parasitology and Ecology, Department of Animal Biology and Physiology, University of Yaoundé I, Yaounde, Cameroon; 5 Center for Research on Genomics and Global Health, National Human Genome Research Institute, National Institutes of Health, Bethesda, MD USA; 6 Wellcome Trust Centre for Global Health Research, Brighton and Sussex Medical School, Falmer Campus, Brighton, BN1 9PX UK

**Keywords:** *Podoconiosis*, *Screening*, *Predictive value*, *Community Health Implementers*, *Cameroon*

## Abstract

**Background:**

The suitability of using clinical assessment to identify patients with podoconiosis in endemic communities has previously been demonstrated. In this study, we explored the feasibility and accuracy of using Community Health Implementers (CHIs) for the large scale clinical screening of the population for podoconiosis in North-west Cameroon.

**Methods:**

Before a regional podoconiosis mapping, 193 CHIs and 50 health personnel selected from 6 health districts were trained in the clinical diagnosis of the disease. After training, CHIs undertook community screening for podoconiosis patients under health personnel supervision. Identified cases were later re-examined by a research team with experience in the clinical identification of podoconiosis.

**Results:**

Cases were identified by CHIs with an overall positive predictive value (PPV) of 48.5% [34.1–70%]. They were more accurate in detecting advanced stages of the disease compared to early stages; OR 2.07, 95% CI = 1.15–3.73, *p* = 0.015 for all advanced stages). Accuracy of detecting cases showed statistically significant differences among health districts (χ2 = 25.30, *p* = 0.0001).

**Conclusion:**

Podoconiosis being a stigmatized disease, the use of CHIs who are familiar to the community appears appropriate for identifying cases through clinical diagnosis. However, to improve their effectiveness and accuracy, more training, supervision and support are required. More emphasis must be given in identifying early clinical stages and in health districts with relatively lower PPVs.

## Background

Access to comprehensive healthcare services of high quality is difficult for socio-economically disadvantaged groups in rural regions. This may be due to the lack of insurance, language barriers or cultural differences [1]. Health service programmes delivered by minimally-trained Community-based Health Implementers (CHIs), also known as Community-based Health Workers (CHWs), have been established in many developing countries [2]. This idea comes from a concept that has been around for at least 50 years [3]. According to a WHO Study Group [4], community health workers should be members of the communities in which they work, should be selected by the communities, should be answerable to the communities for their activities, should be supported by the health system but not necessarily a part of its organization, and have shorter training than professional workers [5, 6]. CHIs may be men or women, young or old, literate or illiterate [3]. Because they are generally indigenous to the communities in which they operate, sharing a common language, ethnicity, socio-economic status, or life experience, CHIs break the culture and language-related barriers between their own community and health care systems. As such, they are perceived by community members to be important sources of information on health issues and on how to access services.

Within the past 20 years, CHIs have been tremendously helpful in many preventive and promotive health programmes such as the African Programme for Onchocerciasis Control (APOC), in which they facilitate drug distribution. In this context, they are often referred to as community drug distributors. Depending on the setting, CHIs are referred to using many appellations: Community Health Volunteers, Village Health Workers, Basic Health Workers, Health Extension Workers, Lay Health Workers, Community Outreach Workers—this list is not exhaustive [3, 6, 7]. In malaria control programs, they have been shown to be very efficient in distribution of Insecticide-impregnated Bed Nets (IBNs) in the community. In addition, CHIs have been intensively used to provide prenatal care, vaccinations, vitamins and check-ups, as well as to promote breastfeeding, oral rehydration, malaria treatment and fever management in remote areas [6, 8, 9]. The majority of these activities require limited diagnosis. Apart from a couple of interventions [10], CHIs have not been intensively used in programmes involving direct clinical screening of the population for a given disease. This may be feasible with diseases (e.g. non-communicable) whose screening approaches are based mostly on physical examination of the patient and to a lesser extent on biomedical examinations.

Podoconiosis is an example of such a disease. It is a geochemical disease mainly characterized by lymphedema which is a condition of localized fluid retention resulting from a compromised lymphatic system [11, 12]. The main clinical feature of podoconiosis, oedema of the foot and lower leg, is similar to that observed in filarial infection due to *Wuchereria bancrofti* [13, 14]. In both diseases, lymphoedema progresses to elephantiasis. However, clinical manifestations such as epididymitis, lymph scrotum, hydrocele and chyluria are more specific to filarial infection [15]. Previous studies have documented the association of podoconiosis with irritant red clay soils, which are generated in areas at 1500 m above sea level (m a.s.l.), with 1000 mm annual rainfall and maximum temperatures of 20°C [12, 16, 17]. The disease has been classified into five stages based on its severity with stage 1 being the least severe stage and stage 5 the most severe stage [18]. Apart from studies carried out by Price and Henderson [19] and Wanji et al. [20] little is known about the geographical distribution of podoconiosis in Cameroon. This study was designed prior to a regional mapping of podoconiosis in North West Cameroon to determine the aptitude and accuracy of CHIs in identifying and staging podoconiosis cases. Clinical examination is a valid means of diagnosing podoconiosis in endemic areas [7, 20]. A previous study in an endemic area in southern Ethiopia using community outreach workers demonstrated a predictive value of up to 100% [7]. The present study was intended to demonstrate the predictive value of CHIs in the diagnosis of podoconiosis in Cameroon.

## Methods

### Study design

This study was designed to determine the feasibility of using Community Health Implementers (CHIs) to map podoconiosis in the North West region of Cameroon. Trained CHIs were employed to carry out a preliminary screening to identify podoconiosis cases in 6 of the 19 health districts of the North West region of Cameroon. In each district, with the aid of the health system, CHIs were selected from among the community drug distributors who had been working for years in the framework of the Community-Directed Treatment with Ivermectin (CDTI). To assess the accuracy of podoconiosis case identification by trained CHIs, all podoconiosis cases presumptively identified (suspected cases) were invited by the research team and re-examined.

### Study site

**Fig. 1:**
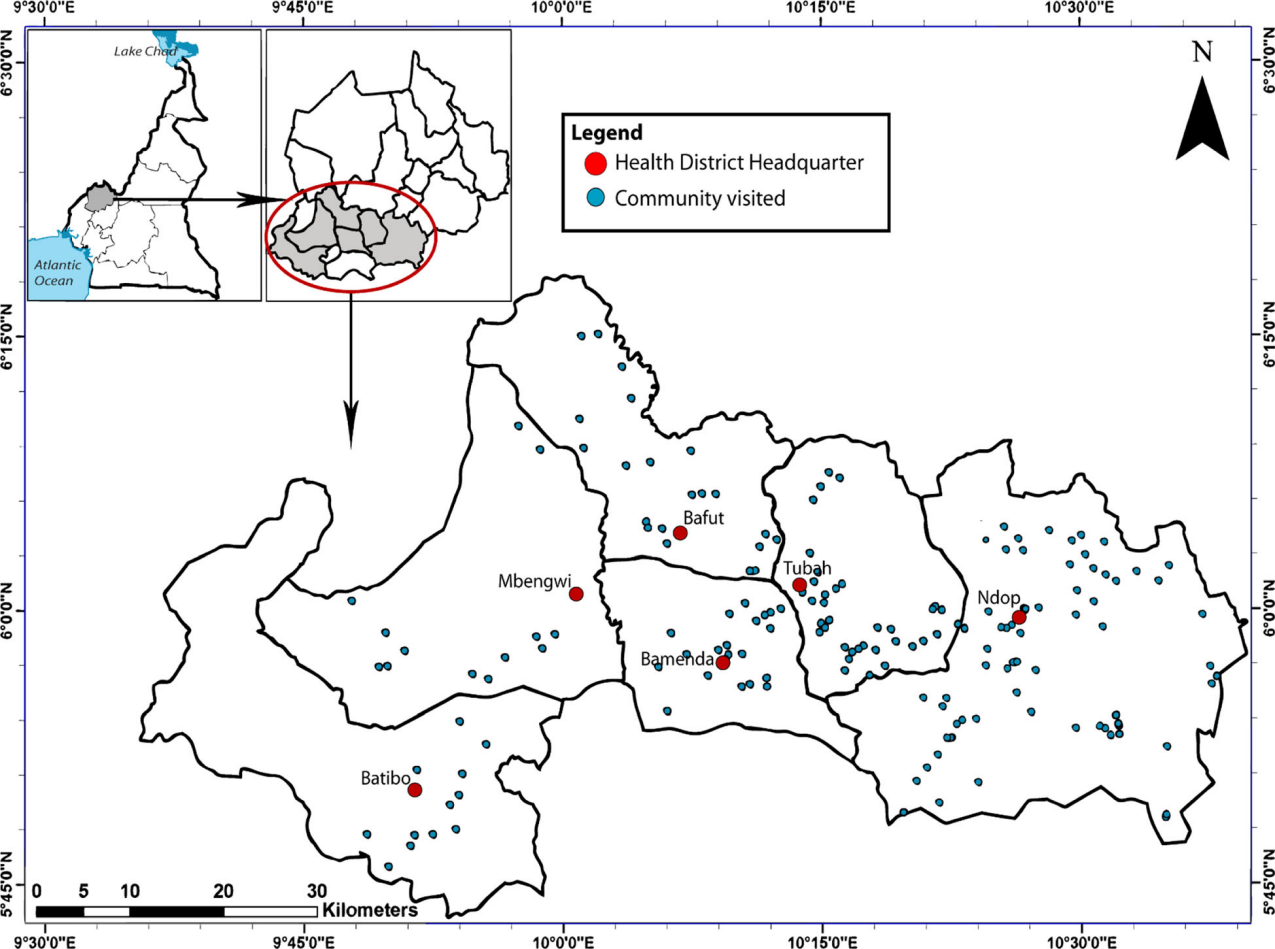
Map of the study site

This preliminary study was carried out in 6 health districts (Bafut, Bamenda, Batibo, Mbengwi, Ndop, Tubah) of the North West region of Cameroon. Nine health areas were selected from both Bafut and Tubah; 4 each from Bamenda, Batibo and Mbengwi and 13 from the Ndop health district (Fig. 1). The presence of podoconiosis had previously been demonstrated in the Ndop and Tubah health districts by Wanji et al [20] hence the selection of the region. The North West region is composed of mostly hilly land with a mean altitude of 1403m above sea level. It experiences two seasons, the dry and the wet, and has a mean annual rainfall of 2500 mm. The very fertile soils in the region are used to grow rice, maize, beans and other vegetables. The main occupation of the population is farming.

### Study population and ethical considerations

The study population was made up of individuals of both sexes and more than 18 years of age who had lived in the area for at least 10 years. This criteria were necessary because podoconiosis is a chronic and debilitating disorder whose onset occurs after at least 10 years’ exposure in endemic areas [21]. Prior to recruitment, the nature, objectives and potential benefits and risks of the study were explained to potential participants, and those who agreed to take part in the study signed a consent form. Participation was voluntary. The approach to the participants was made in accordance with the findings of rapid ethical assessment conducted by Kengne-Ouafo and colleagues [22]. This study was approved by the Cameroon Ethics Committee and the Ministry of Public Health.

### Training of community health implementers

CHIs were invited from all the health areas in each health district. The training was done in 6 different pools (one per health district) by the research team. The trainees were made up of CHIs, health personnel and patients (for demonstration purposes). They were trained on the disease, its causes, clinical manifestations, stages, treatment, prevention and socio-economic impact. During the training, differences between podoconiosis and lymphatic filariasis were explained because many people found it difficult to differentiate between the two diseases. The training also focused on approach to the community and data recording. A practical exercise was conducted with photographs of the different podoconiosis stages including guidelines for their identification.

### Census in health districts, screening of the population for podoconiosis and validation

The census and screening of the population was carried out by CHIs under the supervision of health personnel. CHIs visited households in their quarters or zones, registered all individuals in each household, and did screening for podoconiosis. Research team members who had more experience in the diagnosis of podoconiosis re-examined the cases considered positive by the CHIs.

### Data analysis

A template was created in EPI info v.3.5.3 and the data from registers was entered in it. The data were then cleaned and exported into SPSS version 20 (Chicago, IL, USA) after which point prevalences were computed. A point prevalence is the number of affected people in a health district divided by the total number of people registered, times 100%. The Chi-squared test was used to compare prevalence data or proportions of individuals affected within health districts. CHIs’ and researchers’ findings were compared and an adjustment factor computed. The adjustment factor (positive predictive value) was defined as the ratio of total number of confirmed cases to the total number of suspected cases (present during the validation survey), times 100%. Adjusted prevalence was also computed by multiplying the raw prevalence (obtained through CHIs screening) by the overall predictive value. The logistic regression model was used to measure the association between the accuracy of detecting real cases and disease stage.

## Results

### Training of Community Health Implementers and health personnel

A total of 6 training sessions were organised (one session per health district) during which 50 Chiefs of (health) Centres (COCs) and 193 CHIs were trained. Two CHIs were trained per health area in all Health Districts except in Ndop and Tubah where the number was increased with respect to the number and size of the communities. Table 1 presents the number of health areas, COCs and CHIs trained per health district. COCs and CHIs took part in the patient screening process with COCs playing a supervisory role.

**Table 1:** Participants trained per health district in the North-West region of Cameroon

Health District	Number of Health Areas targeted	No of COCs trained	No of CHIs trained
BAFUT	9	10	18
BAMENDA	4	5	8
BATIBO	4	5	8
MBENGWI	4	5	8
NDOP	14	15	103
TUBAH	9	10	48
TOTAL	42	50	193

*COCs* Chiefs of Centre, *CHIs* Community Health Workers

### CHIs screening results

Of 172 communities selected from 6 Health Districts, a total of 56,479 individuals were registered, among whom 1069 were identified as podoconiosis cases by CHIs giving an overall prevalence of 1.9%. Prevalence ranged from 0.9% in Ndop to 7.7% in Batibo (Table 2). The prevalence was significantly different between districts (χ^2^ = 1023.4 *p* < 0.0001)

**Table 2:** Podoconiosis prevalence within the surveyed Health District

Health District	Communities surveyed	Number registered 20^a^-10^b^ (years)	Podoconiosis cases	Prevalence (%)
BAFUT	23	6077	304	5.1
BAMENDA	21	12,457	134	1.1
BATIBO	12	2918	227	7.7
MBENGWI	11	1865	50	2.7
NDOP	75	25,601	232	0.9
TUBAH	30	7561	122	1.6
Total	172	56,479	1069	1.9

^a^people who were more than 20 years old

^b^those who have lived in the community for more than 10 years

### Researchers’ outcome and relationship between CHIs’ and researchers’ results (Positive predictive value)

During the validation survey, 326 of the 1069 cases previously identified by CHIs were present. Re-examination revealed 158 true podoconiosis cases giving an overall positive predictive value of 48.5%. Predictive values varied between health districts with Ndop registering the highest (70%) and Batibo, the lowest (34.1%), with statistically significant differences (χ^2^ = 25.30, *p* = 0.0001). Among cases identified by the CHIs in the six districts, those in the Bafut, Bameda, and Batibo were at lesser odds of being accurate as validated by the research team compared to those identified in the Ndop district (Table 3).

**Table 3:** Relationship between CHIs’ and researchers’ screening for podoconiosis in North West Cameroon

Health District	Expected cases invited (by CHIs)	Expected cases Present	Confirmed cases	Predictive value^a^ (Based on confirmed cases and number present for validation) (%)	OR (95% CI)	*P*-value
BAFUT	304	86	30	34.9	0.24(0.12–0.56)	<0.0001
BAMENDA	134	45	19	42.2	0.32(0.15–0.70)	0.0034
BATIBO	227	44	15	34.1	0.23(0.10–0.51)	0.0002
BENGWI	50	23	12	52.2	0.48(0.19–1.25)	0.13
NDOP	232	75	52	70.0	Ref	Ref
TUBAH	122	53	30	57.0	0.58(0.28–1.20)	0.14
Overall total	1069	326	158	48.5		

^a^χ2 = 25.30, *P* = 0.0001

### Predictive value with respect to podoconiosis stages

In calculating the predictive value by podoconiosis stage, only 4 health districts were considered, because in Ndop and Tubah, the validation exercise was done globally without taking into account the different stages. The positive predictive value was found to increase with the severity of the disease. It ranged from 31% for stage 2 to 80% for stage 5. Among cases identified by the CHIs those at advanced disease stage were at greater odds of being accurate as validated by the research team than early stage cases (OR = 2.07, 95% CI = 1.15–3.73; *P* = 0.015) (Table 4). Stage 1, being a reversible stage, was not considered in this study.

**Table 4:** Odd of accurately detecting podoconiosis by CHIs taking stage 2 (early disease stage) as reference

Disease stage	Correct	Incorrect	OR (95% confidence interval)	*P*-value
Stage 2	33	75	Ref	Ref
Stage 3	28	33	1.928 [1.01–3.69]	0.047
Stage 4	9	11	1.860 [0.70–4.91]	0.211
Stage 5	4	1	9.091 [0.97–84.47]	0.052
Advanced stages (3,4,5)	41	45	2.07 [1.15–3.75]	0.015

## Discussion

This study was designed to explore the feasibility of using CHIs in interventions necessitating clinical diagnosis of podoconiosis. The work was done prior to the regional mapping of podoconiosis using CHIs to assess how effective and accurate they could be in identifying people with this disease in their communities. CHIs were able to identify podoconiosis cases in the 6 selected health districts. Evaluation of their work revealed a mean positive predictive value of 48.5% (range 34 to 70%). This value is low, and suggests CHIs do not accurately identify patients with podoconiosis in the community. A study carried out by Jacob and colleagues [23] revealed similar results. Trained community health workers diagnosed dementia with low sensitivity and positive predictive values (3.8 and 44.4% respectively).

Desta and colleagues [24] in southern Ethiopia demonstrated clinical diagnosis using Community Podoconiosis Agents (CPAs) to be appropriate in identifying patients in endemic areas where *Wuchereria bancrofti* infection is not suspected. Previous investigations showed the absence of such an infection in our setting [20]. However, the very high predictive value (100%) obtained in Ethiopia could be explained by the fact that the survey was done by more experienced CPAs. The study was indeed carried out in southern Ethiopia, a region where a podoconiosis treatment and prevention association known as Mossy Foot Treatment and Prevention Association (MFTPA) had been operating for over a decade [25]. The MFTPA was organized into clinical and social work sections and operates by transforming treated patients into CPAs. CPAs had good knowledge of the disease and were highly motivated to work with the community. Although the selected CHIs in Cameroon had been working with the community and showed some degree of motivation, they had received their training (one session) just before the screening exercise. This could in part explain the low predictive value obtained. Another study carried out by Gaziano and colleagues [10] showed that trained community health workers (CHWs) could complete screening for cardiovascular diseases risk in a short timeframe with a high level of accuracy (96.8% agreement compared with the gold standard of a health professional). However, it should be noted that in their study, CHWs were trained for 1–2 weeks and only those who met the criteria to do fieldwork were recruited.

It was noticed during the validation process that CHIs often mistook patients with arthritis/rheumatism, varicose veins and diabetic peripheral neuropathy for stage 2 podoconiosis. However, they experienced less difficulty in identifying advanced podoconiosis stages as positive predictive value was seen to increase with disease severity (Table 5). More training in differential diagnosis should therefore be arranged for CHIs and all health personnel who play a supervisory role in many health intervention programmes to circumvent the problem of misdiagnosis of earlier stages precisely in districts. Moreover, training on real patients, particularly with focus on early clinical stages may help in the future. In this study, it was noticed that in two health districts (Tubah and Ndop) where practice during training was done on real patients, the odds of identifying podoconiosis cases correctly was significantly higher compared to other health districts where pictures were used for demonstration purpose. However there was not much significant difference between Ndop and Tubah districts, which were the areas where the 2008 survey was done. This implies that CHIs could have become more familiar with the disease in these districts. It should also be pointed out that a higher number of CHWs were employed in those Health Districts and this could equally explain the high PPVs registered. This observation implies that increasing the number of trained CHW would help to improve the PPV.

**Table 5:** Percent agreement expressed per podoconiosis stage

Severity of the affection	Expected cases invited (by CHIs)	Expected Cases Present	Confirmed cases	Predictive value (Based on confirmed cases and number present for validation) (%)
Stage 2	304	108	33	31
Stage 3	207	61	28	46
Stage 4	59	20	9	45
Stage 5	7	5	4	80

Evaluations of Community Health Worker performance in Kenya found that guideline complexity and inadequate supervision were major inhibitory factors [26, 27]. In addition to adequate supervision, selection and support of CHIs may also constitute a confounding factor for their performance. By definition, CHIs are individuals living in the community where they work and selected by the community to which they are answerable for their activities [4, 10]. However, this is not always the case. Evaluation of some health intervention programmes in some low-income countries revealed that as a rule, local bureaucrats, village chiefs or other dignitaries held sway over who was selected [26, 28]. This is a problem, as selection is often considered a form of patronage. In our study, CHIs were a subset of the Community Drug Distributors who had been working for years with the community in the framework of the mass treatment with ivermectin against onchocerciasis. It is worth mentioning that the CHI subset was selected by the health system with the help of the health committee; so some CHI worked in communities that were not their own, which might have accounted for the low performance seen in some districts.

Overall, many CHIs were happy to do the work, particularly with the training and working materials (umbrella, boots, registers and pens) given to them. However, some complained about the amount of work they were to carry out, the limited length of time that was allocated to do the work and the inadequate remuneration. From discussion with the research team, it appeared that some CHIs who attended the training did not do the work themselves but trained other persons (including their family members) in the community to work for them. The main reason was that they had had a more lucrative job somewhere else hence the need for more support to CHIs. These are some of the factors that might have led to the low predictive value obtained in some areas. In Ethiopia, CPAs are paid a monthly salary by MFTPA [29]. According to Bhattacharyya et al. [30], incentives could be both pecuniary or/and non-pecuniary depending on the setting. The effectiveness of a CHI comes down to his or her relationship with the community. Programmes implementers or the government must do everything they can to strengthen and support this relationship.

Another point to raise is that the approach used by CHIs to screen the community for podoconiosis was different from the one employed by the research team for validation. CHIs’ screening was community-based whereas the validation was clinic-based. Provisionally identified cases were invited to the health centre for re-examination. Some real podoconiosis cases might have been missed by the researchers, those who failed to come to the health centre for comfirmation. Reluctance to attend an external venue may also arise from felt or enacted stigma. Podoconiosis is a stigmatized disease [12, 29, 31], which according to Deribe and colleagues [29] can be classified into felt and enacted. Enacted stigma includes the experience of discrimination such as abuse, loss of employment or prejudicial attitudes, while felt stigma is the perceived fear of enacted stigma. A study carried out in North West Cameroon to explore knowledge, attitudes and perceptions (KAP) relating to lymphoedema demonstrated high levels of stigma with a negative effect on free interaction and acceptance in marriage resulting in many patients staying constantly indoors [20]. A community-based validation may have led to increased CHI predictive value. The use of treated patients by the MFTPA in Ethiopia for social work probably reduces stigma in the community while enhancing mobilization [25].

## Conclusion

This study has demonstrated that CHIs given brief training identify true positive podoconiosis cases although with relatively low accuracy. Podoconiosis being a stigmatized disease, the use of CHIs (people familiar to the community) seems appropriate for the identification of cases. However, to improve the effectiveness and accuracy of CHI diagnosis, more training, supervision and support whether from the intervention programme implementers, the government or the community itself is very necessary. Clear indicators for assessing podoconiosis elimination and endemicity has previously been defined [32, 33].

CHIs involvement could contribute to the rapid determination of the geographical distribution of the disease which in turn would lead to successful targeting of control measures to areas of greatest need.
